# Sublobar Resection of Non-Small-Cell Lung Cancer: Wedge Resection vs. Segmentectomy

**DOI:** 10.3390/curroncol31050187

**Published:** 2024-04-29

**Authors:** Kyeong Ri Yu, Walker A. Julliard

**Affiliations:** Section of Thoracic & Foregut Surgery, Department of Surgery, Virginia Commonwealth University Health System, Richmond, VA 23298, USA

**Keywords:** non-small-cell lung cancer, sublobar, segmentectomy, wedge resection

## Abstract

Lung cancer is the most common cause of cancer death. The mainstay treatment for non-small-cell lung cancer (NSCLC), particularly in the early stages, is surgical resection. Traditionally, lobectomy has been considered the gold-standard technique. Sublobar resection includes segmentectomy and wedge resection. Compared to lobectomy, these procedures have been viewed as a compromise procedure, reserved for those with poor cardiopulmonary function or who are poor surgical candidates for other reasons. However, with the advances in imaging and surgical techniques, the subject of sublobar resection as a curative treatment is being revisited. Many studies have now shown segmentectomy to be equivalent to lobectomy in patients with small (<2.0 cm), peripheral NSCLC. However, there is a mix of evidence when it comes to wedge resection and its suitability as a curative procedure. At this time, until more data can be found, segmentectomy should be considered before wedge resection for patients with early-stage NSCLC.

## 1. Introduction

Lung cancer is one of the most common cancers, with an estimated 238,000 adults diagnosed annually in the United States alone, making it the third most common cancer after breast and prostate. Despite this, it is the most common cause of cancer death; its number of deaths rivals the sum of deaths from breast, prostate, and pancreatic cancer [1].

With a pathology with such high incidence and mortality, lung cancer diagnosis and treatment have long been discussed and studied. The malignancy is divided into two categories—small-cell lung cancer (SCLC) and non-small-cell lung cancer (NSCLC)—based on histology. In recent years, the expansion of the classification of various lung malignancies has led to more specific targeted therapy for each lung cancer in terms of systemic immunotherapy, chemotherapy, and radiation [2]. However, this extensive and detailed classification has not been employed in depth for surgical management. Patients with either SCLC or NSCLC, but more so the latter, can be candidates for surgical resection, with the TNM system serving as the mainstay guideline.

## 2. Workup

Lung cancer can be discovered in patients with suggestive symptoms undergoing a workup, via screening in appropriate patients, or incidentally on imaging obtained for another reason. Common symptoms of lung cancer include a cough, dyspnea, and chest pain [3]. A combination of imaging modalities is used when investigating a suspected case of lung cancer. Chest radiography, while inadequate for staging and further characterization, provides preliminary information about the disease. Contrast-enhanced chest computer tomography (CT) is the foundation of lung cancer management and helps to determine the tumor, node, and metastasis (TNM) staging. The goal of the imaging is to define the primary lung tumor in its size, location, and invasion for the T stage. The regional lymph node involvement is also assessed in its laterality and location for the N stage. Lastly, imaging evaluates distant metastasis, specifically looking for an extension to the pleura and pericardium, as well as to the chest wall, nearby organs such as the liver and adrenal glands, and the soft tissues [4].

It is important to highlight that a lung cancer diagnosis must only be made with definitive pathology in the form of tissue biopsy, whether bronchoscopic or image-guided. Bronchoscopy with endobronchial ultrasound (EBUS)-directed biopsy has risen recently as a common modality for diagnosing and staging suspected NSCLC [5]. Based on the location, percutaneous image-guided biopsy is also utilized for lesions less accessible by bronchoscopy. For lesions that are small and isolated, surgical biopsy is preferred as it can be both diagnostic and curative.

Imaging is sufficient for mediastinal staging if no mediastinal abnormalities are seen and the risk of N2 is low—but if sampling is indicated, similarly to tissue biopsy, EBUS-guided transbronchial needle aspiration and endoscopic ultrasound-guided fine needle aspiration can be utilized. For selected patients with negative results, cervical mediastinoscopy serves as a more invasive, but highly sensitive (80%) and specific (100%) procedure [6,7].

With information obtained from imaging, laboratory tests, pathology, and clinical history, clinical staging can be performed to facilitate the choice of the optimal therapy. The International Association of the Study of Lung Cancer (IASLC) International Staging Project released the 8th edition of TNM classification, which determines each T, N, and M descriptor, then able to be grouped into stages 0–IVB [4].

## 3. Management

With SCLC more than often presenting as a disseminated disease, the disease is most often managed with systemic therapy. Surgery is reserved for select patients with limited-stage disease, which equates to a single pulmonary nodule and regional lymph node involvement without distant metastases [8]. On the other hand, surgery plays an integral role in the treatment plan for NSCLC.

Surgery is usually the initial treatment for early-stage NSCLC, including stages I and II. This includes patients whose tumors measure less than 7 cm with no regional lymph node involvement or distant metastasis. As the tumor is contained within the lung, the surgery entails complete resection of the tumor and its draining lymph nodes. As per IASLC, complete resection must include negative resection margins, systemic nodal dissection or lobe-specific systemic nodal dissection, and no extracapsular or distal mediastinal nodal extension [9].

## 4. Methodology

This review was conducted using the following databases: PubMed, ScienceDirect, Cochrane Library, and Scopus. The aim was to collect publications discussing the subject of sublobar resection including segmentectomy and wedge resection, particularly those comparing sublobar resection with lobectomy for early-stage non-small-cell lung cancer. The included studies fall under the following criteria: (1) the publication is in English, or provides a translation into English language; (2) the subjects included in the study have early-stage or stage I non-small-cell lung cancer; (3) the study was designed to compare sublobar resection and lobectomy in the form of a retrospective cohort study, randomized controlled trial, or meta-analysis; and (4) the results discuss at least one outcome, such as overall survival or cancer-specific survival. All studies reviewed included human subjects; animal studies were excluded. This article serves as a review and a cumulation of relevant studies; no statistical analysis was performed (Table 1, Table 2, Table 3 and Table 4). We hope to provide an in-depth discussion of each article and present current evidence regarding sublobar resection for early-stage NSCLC in a comprehensive, easy-to-peruse fashion, especially for those who may be less familiar with the topic.

## 5. Surgical Techniques

Historically, surgical removal of lung carcinoma was long deemed to be impossible due to its high mortality. Before the 1900s, as general anesthesia was limited only to inhalational agents, the key to successful lung surgery was to be fast, before the resultant pneumothorax from the thoracotomy caused paradoxical breathing, hypoxia, and death. Positive-pressure ventilation was first described in 1909, and it took another 20 years before selective one-lung ventilation was described, which led to monumental progress in thoracic surgery. After the first pneumectomy was performed in 1933, it remained the mainstay treatment for lung cancer until 1962, when the landmark case series by Shimkin et al. showed that lobectomy was a non-inferior equivalent to pneumectomy, with fewer complications. Thus, lobectomy became the gold-standard treatment for lung resection [34]. Sublobar resection (SLR), which resects less than a whole lobe in its entirety, was explored by many surgeons. However, in 1995, the Lung Cancer Study Group published a prospective randomized controlled trial that demonstrated a significant increase in local recurrence and a decrease in overall survival (OS) in the SLR group compared to lobectomy [10]. The study had its criticisms, including disparities in preoperative workups amongst patients as well as erroneous data interpretation, but it solidified the gold-standard status of the lobectomy for early-stage NSCLC, leaving sublobar resection to be a compromise procedure reserved for patients who could not tolerate lobectomy [9,34]. However, the advances in CT over the years have led to earlier detection and staging of NSCLC. Sublobar resection has now regained attention as a treatment for these smaller lung tumors. This review article inspects current evidence dating back to 1995 for the role of sublobar resection in NSCLC, including both wedge resection and segmentectomy.

## 6. Sublobar Resection

Sublobar resection can be performed either as anatomical segmentectomy or nonanatomical wedge resection. Segmentectomy begins with a hilar dissection, similar to a formal lobectomy, and dissection continues following bronchovascular structures. In this way, the three key structures of each segment (the artery, vein, and bronchus) are identified and transected. Essentially, this dissection leads to significant nodal harvest from regional N1 nodes. The parenchymal division then follows to complete the segmentectomy [34,35]. Wedge resection, on the other hand, involves stapling directly across the lung parenchyma to resect the portion with the tumor of interest. The advantage of wedge resection is that it preserves a larger lung reserve than other surgical options, thus having been reserved for patients with poor baseline pulmonary function or significant comorbidities. This was demonstrated in a study by Chiang, which showed that propensity-matched patients undergoing wedge resection had better perioperative outcomes including shorter operative time, less blood loss, and a shorter hospital stay with a shorter duration of chest tube presence [29]. At the same time, there have been concerns that wedge resection removes less parenchyma and fewer lymph nodes than lobectomy or segmentectomy, raising concerns about a worse overall outcome.

## 7. Segmentectomy vs. Lobectomy

As segmentectomy removes less lung parenchyma than lobectomy, it is logical to infer that the former will preserve more lung function. Several studies have demonstrated this: a retrospective study by Nomori showed that segmentectomy results in the preservation of whole lung function in the operated lobe as opposed to lobectomy [18]; another study by Keenan demonstrated at 1 year postoperatively that lobectomy patients experienced significant declines in pulmonary function tests compared to segmentectomy patients, with the 1-year survival rate similar in both groups [15].

Several meta-analysis studies have been published comparing segmentectomy to lobectomy in early-stage NSCLC. In 2012, Fan et al. reported that there was a significant benefit of lobectomy over sublobar resection in OS and cancer-specific survival (CSS) among stage I NSCLC patients, but this significance was not observed in the same group when segmentectomy was isolated and directly compared with lobectomy. While it was not directly stated, this analysis implied that the inclusion of wedge resection in sublobar resection may contribute to its inferiority to lobectomy [12]. Another study a few years later, in 2017, compared lobectomy with segmentectomy alone, which was shown to be non-inferior in overall survival in stage I NSCLC patients [16]. A meta-analysis study by Winckelmans et al. in 2020 reviewed 28 studies that targeted patients with stage I and stage IA NSCLC. The included studies were a mix of retrospective studies with one nonrandom prospective study and one case-matched study. Unlike the former two studies, this study highlighted the difference in statistical significance by stratifying the patient population into those with stage I, stage IA, or stage IA and with a tumor size <2 cm. It concluded that segmentectomy is inferior to lobectomy in stage I and stage IA, but not in stage IA < 2 cm, narrowing down the patient criteria appropriate for segmentectomy [19]. This recommendation was further reinforced by another study published this year by Dai et al., which included 10 articles that focused on perioperative outcomes and survival, and demonstrated that lobectomy was superior to segmentectomy in the entire stage IA NSCLC population in OS and disease-free survival (DFS), as well as the stage IA 2–3 cm subgroup. The OS outcomes were similar between lobectomy and segmentectomy in the stage IA < 2 cm subgroup [23].

The Japan Clinical Oncology Group (JCOG) and the West Japan Oncology Group (WJOG) then performed three prospective multi-institutional studies to compare sublobar resection and lobectomy, with a greater focus on segmentectomy in two of the three studies. JCOG0802/WJOG4607L was the first randomized phase 3 trial since the 1995 landmark trial to revisit the non-inferiority of sublobar resection, specifically segmentectomy. Over 1100 patients with stage IA < 2 cm were randomly assigned between segmentectomy and lobectomy. The results showed that the 5-year overall survival in the segmentectomy group was both non-inferior and superior to the lobectomy group; the total relapse pattern was similar in the two groups—suggesting that segmentectomy should be standard in such a specific population [20]. While the JCOG0802 trial focused on patients with solid tumors 2 cm or smaller in order to directly compare segmentectomy with lobectomy, the JCOG1211 trial looked at patients with ground glass opacity (GGO) up to 3 cm in size undergoing segmentectomy. The study showed that as long as a sufficient surgical margin was secured, segmentectomy was a suitable curative treatment with good preservation of postoperative pulmonary function in patients with GGO up to 3 cm [22]. Around a similar time, Stamatis et al. also independently performed a prospective, randomized, multicenter phase 3 trial to compare segmentectomy and lobectomy, with patients with suspected or verified NSCLC up to 2 cm. It demonstrated no statistical differences in perioperative outcomes, either in terms of the locoregional or distant metastases or OS. It also showed the non-inferiority of segmentectomy to lobectomy in DFS [21].

While many of these studies mentioned above acknowledge the preliminary nature of their findings, the common consensus is that tumor size is a determining factor in survival and recurrence in utilizing segmentectomy for patients with early NSCLC.

## 8. Wedge Resection vs. Lobectomy

One of the controversies behind the 1995 study by the Lung Cancer Study Group mentioned above is that a significant portion of the sublobar resection was wedge resection, at 32.8%, as raised by the 2006 multicenter study by Okada et al. The latter study infers that the predominance of wedge resection in the 1995 study may have affected the local recurrence seen in the sublobar group. It also demonstrated that in a combination of segmentectomy and wedge resection, the percentage of the latter procedure was much smaller at 9.8%, and there were no significant differences in morbidity, curability, or recurrence rate with similar OS and DFS between sublobar resection and lobectomy for patients with cT1N0M0 NSCLC [11].

With the efficacy of wedge resection as an oncological procedure in question, McGuire et al. sought to compare directly between wedge resection and lobectomy in terms of recurrence and survival in a retrospective study, and determined that when limited to selected patients with smaller tumors under 2 cm and diagnosed with an earlier stage such as stage IA and IB, the disease-free and overall survival were similar in both groups [24]. However, another study retrospectively reviewed patients with stage T1 tumors undergoing wedge resection and lobectomy and found that the former had significantly reduced 5-year survival rates. The important distinction is that the study in question did not acknowledge tumor size when propensity-matching their patients, which may have affected the results [25].

## 9. Segmentectomy vs. Wedge Resection

The question remains—is wedge resection comparable to segmentectomy or should it be limited to an even more specific subset of the patient population with early NSCLC? The 2008 study by Sienel directly compared the two sublobar resection techniques. It affirmed the idea that segmentectomy is superior to wedge resection, by demonstrating that patients with stage IA NSCLC undergoing segmentectomy had significantly fewer locoregional occurrences and better cancer-related survival than those who underwent wedge resection [26]. On the other hand, the retrospective review by Altorki showed that while more patients underwent nodal sampling during segmentectomy with more stations sampled and total nodes resected, there was no difference in local recurrence or 5-year DFS between the two procedures [27]. More recently, as part of a series evaluating the efficacy and safety of sublobar resection, the nonrandomized confirmatory phase 3 JCOG0804 trial enrolled patients with GGO-dominant lung cancer, with a heavier emphasis on wedge resection—only enrolling patients in segmentectomy when surgical margins were insufficient. The 5-year relapse-free survival (RFS) was 99.7%, with no local relapse; at the 10-year mark, the RFS and OS were 98.6% and 98.5%, with only one local recurrence, suggesting that sublobar resection, even when wedge resection is dominant, can be curative [13,36].

Some studies delved into the subject with a more specific subset of patients with NSCLC. Chiang et al. in Taiwan enrolled patients with cT1N0 lung adenocarcinoma and compared the clinical outcomes between those who underwent wedge resection and those who underwent segmentectomy. Despite the better perioperative outcomes seen in the former group, the latter was found to be associated with better DFS in patients with a tumor size greater than 2 cm and consolidation-to-tumor ratio (CTR) higher than 50% [29]. Similarly, Bachman et al. identified patients with clinical T1N0M0 typical bronchopulmonary carcinoid tumors who underwent wedge resection or segmentectomy. The mean tumor size for these patients was below 2 cm: 1.4 cm for the former and 1.7 cm for the latter. The study showed that in these patients, survival rates were similar for the two procedures, and that wedge resection can be considered an alternative for these patients [30]. Of note, while bronchopulmonary carcinoid tumors are neuroendocrine malignancies classified separately from NSCLC, the majority of these tumors are treated through surgical resection, much like early-stage NSCLC [37].

Some meta-analyses have been performed to directly compare lobectomy, segmentectomy, and wedge resection. In 2018, Cao et al. used the Surveillance, Epidemiology, and End Results (SEER) database to directly compare the three techniques, with the two sublobar resection techniques separated as independent groups. Notably, patients were matched in cohorts of tumor size 1.0 cm or smaller, 1.1 to 2.0 cm, and 2.1 to 3.0 cm. When propensity-matched, all three procedures had similar OS and LCSS in the <1 cm group, but OS and LCSS were lower in wedge resection compared to lobectomy and segmentectomy in the 1.1–2.0 cm group. Lastly, lobectomy proved to be superior in both outcomes for tumors of 2.1 cm or larger. These results suggested that wedge resection is a comparable procedure for tumor sizes of 1.0 cm or smaller but not necessarily for larger tumors [37]. Another meta-analysis by Shi similarly compared all three procedures and found similar results, with the OS for lobectomy better than that of wedge resection and with similar DFS and RFS. However, this study did not take tumor size into account for its Bayesian analysis [31]. Amongst those comparing segmentectomy and wedge resection, the most recent and perhaps most inclusive review in the number of studies so far is the meta-analysis by Lin et al., published in early 2024, which analyzed 26 studies to compare sublobar resection techniques with lobectomy for patients with solid-dominant stage IA lung cancer. The authors noted that there was a significant heterogeneity in the studies they accumulated. Otherwise, they found that while OS between sublobar resection and lobectomy was not statistically significant, lobectomy was associated with better RFS overall. When these results were pooled with different populations taken into account, sublobar resection was associated with an OS for a tumor size < 2 cm similar to lobectomy, but it was lower if the tumor size was 2–3 cm. RFS was found to be lower in sublobar resection than lobectomy regardless of tumor sizes. Furthermore, when broken down into different surgical techniques, segmentectomy provided similar OS and RFS to lobectomy across different tumor sizes, whether it be <2 cm or 2–3 cm. Wedge resection, on the other hand, was shown to have lower OS and RFS for larger tumor sizes, and lower RFS in tumors <2 cm. The data were consistent with the other two meta-analyses: the chance of oncologic outcomes comparable to lobectomy inversely depended on the size of the tumor [33].

Given the literature evidence above, it would not be inappropriate to infer that wedge resection should only be performed after a careful evaluation of patient characteristics and their cancer progression, most importantly limiting the procedure to tumor sizes of 1.0 cm or smaller. However, in 2023, Altorki et al. published their own randomized phase 3 trial CALGB140503, comparing sublobar resection and lobectomy, with wedge resection occupying 59.1% of the sublobar resection group. This was a 10-year-long study with a total of 697 patients and a median follow-up of 7 years. Patients with histologically confirmed NSCLC <2 cm were recruited from a wide breadth of institutions in three countries: the United States, Canada, and Australia. The trial also required confirmation of N0 status via preoperative nodal sampling or frozen-section examination. Patients were randomized to either sublobar resection or lobectomy; the type of sublobar resection (between wedge resection and segmentectomy) was at the surgeon’s discretion. What separates this study from the JCOG0802 trial is the inclusion of wedge resection, as is frequently performed in North America and Europe, making this trial more generalizable to the global population. Despite that the majority of the sublobar resection consisting of wedge resection, the results showed that both DFS and OS were similar between the sublobar resection and the lobectomy group, indicating that the former is non-inferior to the latter. Furthermore, the post hoc analysis published months later revealed that all three procedures (wedge resection, segmentectomy, and lobectomy) had similar survival outcomes and locoregional recurrences, despite over 90% of the patients undergoing each procedure having a tumor size of 1.0 cm or larger. Overall, while it is only one trial, the significance of these results challenges the notion that wedge resection is a suboptimal procedure [14,32].

## 10. Discussion

Medicine is constantly evolving. Despite the landmark trial that deemed sublobar resection to be inferior to lobectomy, many studies have now shown that sublobar resection, in an appropriate patient population, is a suitable alternative to lobectomy with similar survival outcomes. This is due to the continued advances in imaging, treatment options, and surgical techniques. However, many of the studies discussed in this review combine wedge resection and segmentectomy under one umbrella group of sublobar resection, with segmentectomy as the more common procedure of the two. The JCOG0802 and JCOG1211 trials have demonstrated the non-inferiority of segmentectomy when compared to lobectomy in both small peripheral NSCLC and GGO. There is much more left to be desired before we can say the same about wedge resection. There are some factors that can be studied more in-depth, in terms of its superiority in perioperative outcomes, specifically the length of the surgery, the length of postoperative stay, the number of anesthesia or surgical complications, and patient comfort levels. Wedge resection has been shown to preserve postoperative lung functions with minimal loss—its strength could be further explored in comparison to lobectomy. While the role of wedge resection as the compromise procedure for patients with comorbidities and limited lung function has been established, its status as a curative procedure for NSCLC has sizeable room for doubt. Several retrospective reviews have shown that in the right population, wedge resection could be as suitable as segmentectomy. This is now further bolstered by the most recent CALGBG140503 trial by Altorki, which showed that all three techniques are equivocal in their outcomes. Of course, that study is not free of its limitations. The population was highly specific, limited to patients with NSCLC of 2 cm or smaller and with no evidence of nodal disease. Given that many wedge resections are performed with minimal to no nodal sampling, it cannot be easily generalized. The authors themselves also acknowledge that the study itself was not designed or powered to test for the non-inferiority of wedge resection to segmentectomy. But this is one of the first major randomized trials that includes wedge resection in significant numbers, making this perhaps the best evidence so far that supports the surgery as an oncological treatment of choice. The promising outcomes from this trial may lead to further exploration and, in the future, even the establishment of wedge resection as another standard procedure for early-stage NSCLC.

## 11. Limitations

A literature review was performed primarily through PubMed with a focus on keywords including “non-small-cell lung cancer”, “wedge resection”, “segmentectomy”, “lobectomy”, and “early-stage NSCLC”. Given the nature of review articles, this manuscript does not capture all studies on wedge resection and segmentectomy, despite our best efforts, and may be subject to selection bias.

An important point to make is that the patient population in most of these studies was limited to those with node-negative diseases. Many elected to confirm the nodal status intraoperatively by sending a frozen section for analysis, but if the preoperative workup included mediastinoscopy or EBUS was performed, no additional lymph node sampling was needed. However, there was inconsistency across these studies: some mandated that all lobectomy patients undergo systematic lymph node dissection, with stations varying by laterality and the lobe where the nodule was located, while sublobar resection patients underwent nodal sampling; and some indicated that both segmentectomy and lobectomy patients received hilar and mediastinal lymph node dissection, ideally systematic but acceptably selective. The treatment for patients found to have nodal metastasis also varied: Suzuki terminated the protocol, but in Aokage’s trial, conversion from segmentectomy to lobectomy was allowed [13,22]. Patients undergoing wedge resection tended to have significantly fewer lymph nodes excised than segmentectomy or lobectomy patients due to the nature of the surgical technique, which forgoes systematic dissection in favor of lymph node sampling instead. The majority of the studies included in this review did not delve deep into the effect of lymphadenectomy on overall survival and recurrence. For studies that did, such as Qu et al., the evaluation of lymph nodes was seen independent of outcomes between segmentectomy and lobectomy [17]. Sienel et al. found that when propensity-matched, the number of lymph nodes excised did not impact the prognostic effects between the two techniques of sublobar resection [26]. Lin’s meta-analysis offered the suggestion that segmentectomy allowed for more thorough lymph node evaluation than wedge resection, but it did not include relevant data in the quantitative analysis to support that suggestion [33]. The CALGB140503 trial by Altorki employed intraoperative confirmation of the node-negative status and accepted simple sampling, systematic sampling, or complete dissection in any of the surgical techniques [14]. The wedge resection group was more likely to include simple sampling or mediastinal dissection compared to the lobectomy group, but all patients included in the study at least had one major hilar or two mediastinal lymph node stations sampled [32]. While the data specific to lymph node evaluation so far may be lacking in their consistency, they still add to the hope that, as per the results produced in Altorki’s newest trial, wedge resection, even with less extensive lymph node evaluation, may be as oncologically acceptable as segmentectomy or lobectomy.

## Figures and Tables

**Table 1:** Comparison studies between lobectomy and sublobar resection overall.

Author	Year	Ref	Study Design	Title
Ginsberg et al.	1995	[10]	RCT	Randomized trial of lobectomy versus limited resection for T1 N0 non-small cell lung cancer. Lung Cancer Study Group
Okada et al.	2006	[11]	NCS	Radical sublobar resection for small-sized non-small cell lung cancer: a multicenter study.
Fan et al.	2012	[12]	Meta-analysis	Sublobectomy versus lobectomy for stage I non-small-cell lung cancer, a meta-analysis of published studies
Suzuki et al.	2017	[13]	NCS	A non-randomized confirmatory phase III study of sublobar surgical resection for peripheral ground glass opacity dominant lung cancer defined with thoracic thin-section computed tomography (JCOG0804/WJOG4507L)
Altorki et al.	2023	[14]	RCT	Lobar or Sublobar Resection for Peripheral Stage IA Non-Small-Cell Lung Cancer

RCT: randomized controlled trial; NCS: nonrandomized cohort study.

**Table 2:** Comparison studies between lobectomy and segmentectomy only.

Author	Year	Ref	Study Design	Title
Keenan et al.	2004	[15]	RCS	Segmental resection spares pulmonary function in patients with stage I lung cancer
Bedetti et al.	2017	[16]	Meta-analysis	Segmentectomy versus lobectomy for stage I non-small cell lung cancer: a systematic review and meta-analysis
Qu et al.	2017	[17]	RCS	Long-term outcomes of stage I NSCLC (≤3 cm) patients following segmentectomy are equivalent to lobectomy under analogous extent of lymph node removal: a PSM based analysis
Nomori et al.	2018	[18]	RCS	Differences in postoperative changes in pulmonary functions following segmentectomy compared with lobectomy
Winckelmans et al.	2020	[19]	Meta-analysis	Segmentectomy or lobectomy for early-stage non-small-cell lung cancer: a systematic review and meta-analysis
Saji et al.	2022	[20]	RCT	Segmentectomy versus lobectomy in small-sized peripheral non-small-cell lung cancer (JCOG0802/WJOG4607L): a multicentre, open-label, phase 3, randomised, controlled, non-inferiority trial.
Stamatis et al.	2022	[21]	RCT	Survival outcomes in a prospective randomized multicenter Phase III trial comparing patients undergoing anatomical segmentectomy versus standard lobectomy for non-small cell lung cancer up to 2 cm.
Aokage et al.	2023	[22]	NCS	Segmentectomy for ground-glass-dominant lung cancer with a tumour diameter of 3 cm or less including ground-glass opacity (JCOG1211): a multicentre, single-arm, confirmatory, phase 3 trial.
Dai et al.	2023	[23]	Meta-analysis	Systematic review and meta-analysis of segmentectomy vs. lobectomy for stage IA non-small cell lung cancer

RCS: retrospective cohort study; NCS: nonrandomized cohort study.

**Table 3:** Comparison studies between lobectomy and wedge resection.

Author	Year	Ref	Study Design	Title
McGuire et al.	2013	[24]	RCS	Outcomes: wedge resection versus lobectomy for non-small cell lung cancer at the Cancer Centre of Southeastern Ontario 1998–2009
Moon et al.	2023	[25]	RCS	Wedge resection versus lobectomy in T1 lung cancer patients: a propensity matched analysis

RCS: retrospective cohort study.

**Table 4:** Comparison studies between segmentectomy and wedge resection, with or without comparison with lobectomy.

Author	Year	Ref	Study Design	Title
Sienel et al.	2008	[26]	RCS	Sublobar resections in stage IA non-small cell lung cancer: segmentectomies result in significantly better cancer-related survival than wedge resections.
Altorki et al.	2016	[27]	RCS	Anatomical Segmentectomy and Wedge Resections Are Associated with Comparable Outcomes for Patients with Small cT1N0 Non-Small Cell Lung Cancer
Cao et al.	2018	[28]	Meta-analysis	Survival Rates After Lobectomy, Segmentectomy, and Wedge Resection for Non-Small Cell Lung Cancer
Chiang et al.	2021	[29]	RCS	Thoracoscopic Wedge Resection Versus Segmentectomy for cT1N0 Lung Adenocarcinoma
Bachman et al.	2022	[30]	RCS	Wedge Resection Offers Similar Survival to Segmentectomy for Typical Carcinoid Tumors
Shi et al.	2022	[31]	Meta-analysis	Comparison Between Wedge Resection and Lobectomy/Segmentectomy for Early-Stage Non-small Cell Lung Cancer: A Bayesian Meta-analysis and Systematic Review
Altorki et al.	2023	[32]	RCT	Lobectomy, segmentectomy, or wedge resection for peripheral clinical T1aN0 non-small cell lung cancer: A post hoc analysis of CALGB 140503 (Alliance).
Lin et al.	2024	[33]	Meta-analysis	Differential efficacy of segmentectomy and wedge resection in sublobar resection compared to lobectomy for solid-dominant stage IA lung cancer: a systematic review and meta-analysis.

RCS: retrospective cohort study; RCT: randomized controlled trial.
